# Effectiveness of Educational Interventions to Increase Knowledge of Evidence-Based Practice Among Nurses and Physiotherapists in Primary Health Care: Protocol for a Systematic Review

**DOI:** 10.2196/17621

**Published:** 2020-11-02

**Authors:** Henk Verloo, Pauline Melly, Roger Hilfiker, Filipa Pereira

**Affiliations:** 1 Service of Old Age Psychiatry University Hospital of Lausanne Sion Switzerland; 2 School of Health Sciences Haute Ecole Spécialisé Suisse Occidentale Valais/Wallis Sion Switzerland; 3 Institute of Biomedical Sciences Abel Salazar University of Porto Porto Portugal

**Keywords:** evidence-based practice, primary healthcare, beliefs, knowledge, implementation, nurses, nurse practitioners, physiotherapists, interventions, education

## Abstract

**Background:**

The implementation of evidence-based practice (EBP) in daily health care practice is strongly encouraged; it is widely recognized as a means to improve the quality and safety of health care for patients and reduce avoidable costs. Primary care nurses and physiotherapists face numerous challenges in trying to ensure that they deliver effective daily care. Broadly promoted educational interventions aim to increase the integration and implementation of EBP in their daily practice.

**Objective:**

This systematic review will retrieve and evaluate publications examining the effectiveness of educational interventions to increase the integration and implementation of EBP among nurses, nurse practitioners, and physiotherapists active in primary care.

**Methods:**

We will conduct a systematic review of published articles in relevant professional, scientific journals (from their start dates) and in the following electronic databases, from inception until October 31, 2020: Medline Ovid SP (from 1946), PubMed (NOT Medline[sb]; from 1996), Embase.com (from 1947), CINAHL Ebesco (from 1937), the Cochrane Central Register of Controlled Trials Wiley (from 1992), PsycINFO Ovid SP (from 1806), Web of Science Core collection (from 1900), PEDro (from 1999), the JBI Database of Systematic Reviews and Implementation Reports (from 1998), and the Trip Database (from 1997). We will use the predefined search terms of “evidence-based practice,” “nurses,” or “physiotherapists” and combinations with other terms, such as “educational interventions.” We will also conduct a hand search of the bibliographies of all the relevant articles and a search for unpublished studies using Google Scholar, the ProQuest Dissertations and Theses dissemination, Mednar, WorldCat, OpenGrey, and Grey Literature Report. We will consider publications in English, French, German, and Portuguese.

**Results:**

The electronic database searches were completed in October 2020. Retrieved articles are currently being screened, and the entire study is expected to be completed by March 2021.

**Conclusions:**

This systematic review will provide specific knowledge about the effectiveness of educational interventions to increase the implementation and integration of EBP in the daily practice of nurses and physiotherapists providing primary care services. Its findings will inform us about the types and frequencies of the most successful educational interventions.

**Trial Registration:**

PROSPERO International Prospective Register of Systematic Reviews CRD42017077309; https://www.crd.york.ac.uk/prospero/display_record.php?RecordID=77309

**International Registered Report Identifier (IRRID):**

DERR1-10.2196/17621

## Introduction

Evidence-based practice (EBP) is an emerging, breakthrough approach among health care providers (HCPs) [1,2]. It has its origins in evidence-based medicine, which has been defined as “the conscientious and judicious use of current best evidence in making decisions about the care of individual patients” [3]. Many evidence-based models were born of the evidence-based medicine model and helped understand how this concept could be applied to other health professions [4]. One of the ways in which EBP was first conceptualized in nursing was through its use in research. Although EBP includes a patient-centered approach, in research it is simply the rigorous use of research steps to critically appraise research evidence and implement that evidence in practice [5,6].

HCPs are expected to use EBP as a standard approach to daily practice [7-9], integrating research, patient preferences, clinical expertise, and innovative technologies [10,11]. However, the implementation of EBP remains a controversial process [12,13], and not all HCPs are convinced that it improves the quality of care [14,15]. Implementing EBP is challenging, especially in primary health care settings [16,17]. The Swiss Federal Law on Healthcare Professionals will change in 2020 [18]. All health care professionals active in Swiss health care settings will be expected to implement evidence-based care and treatments in their daily practice. Bearing in mind that not all health care professionals received training about EBP during their career trajectory, this raises questions about which educational interventions are most effective at increasing EBP skills among nurses and physiotherapists (PTs) in daily practice. Numerous studies have investigated perceptions about EBP among a variety of health care professionals [9,19,20]. Overall, most of them had positive attitudes towards EBP but lacked the knowledge and skills to implement it. A number of personal and organizational barriers impede EBP implementation [21].

This systematic review will support this reflection and examine those educational strategies. We expect this project to inspire other university hospitals and training centers for allied health care professionals to integrate creative and effective educational strategies to increase EBP skills.

Primary health care is defined as the entry level into a health care services system [22], providing the first point of contact for all new needs and problems. It involves patient-focused care over time, care for all but the most uncommon or unusual conditions, and coordination or integration of that care, regardless of where or by whom it is delivered. It is the primary means by which to approach the main goal of any health care services system: optimization of health status [23]. Health care provided by primary HCPs includes health promotion, prevention and diagnosis, detection, intervention, treatment, and case and care management [24,25]. Furthermore, primary HCPs, especially community health care nurses and PTs, are highly involved in frontline health care services to home-dwelling adult patients and long-term nursing home patients [26,27].

Nevertheless, in some acute health situations, home-dwelling individuals will need to be referred to medical specialists or acute hospital services for additional health care advice. Because of their close relationships with health care users during their daily practice, community health care nurses and PTs play important decision-making roles, strengthening communication and collaboration between the community and specialized HCPs in order to provide the best available overall health care to community-dwelling individuals [28]. Although it is generally considered that community health care nurses and PTs, just like all other HCPs, are accountable for providing the best available evidence-based health care [29,30], recent research has concluded that only a small percentage of them consistently do so [8]. EBP implementation rates among nurses and PTs in hospital institutions have been extensively documented [31-33], and multiple barriers to implementation have been reported [34,35]. These include time constraints, negative attitudes and a lack of personal motivation, professional resistance to research, and inadequate knowledge of and skills for EBP among clinicians [8,36,37].

Additionally, several authors have documented administrative and organizational problems in the workplace, a lack of mentors for EBP, inadequate resources at the point of care, gaps between theory and practice, the lack of any meaningful transition between training courses on EBP and the clinical reality, and an absence or lack of basic education on the subject [38-40]. Finally, different authors have highlighted that HCPs’ beliefs about EBP are associated with their capacity to implement it [31,41,42]. Over the last 2 decades, the use of EBP in health care has been documented in exploratory and observational studies in different settings. Scurlock-Evans et al [8] summarized attitudes, barriers, enablers, and EBP interventions among PTs, although without specifying employment settings or assessing educational interventions. Melender et al [43] summarized the educational interventions used to train nursing students to improve outcomes in the implementation of EBP. Nevertheless, to the best of our knowledge, there has been no systematic review examining the effectiveness of educational interventions aimed at increasing the use of EBP in daily practice among nurses, nurse practitioners (NPs), and PTs active in primary health care.

Our research question is: How effective are educational interventions to increase the implementation of EBP in the daily practice of nurses and PTs delivering primary care among community-dwelling adults?

## Methods

This review will be conducted following the Preferred Reporting Items for Systematic Reviews and Meta-Analyses for Protocols (PRISMA-P) recommendations [44], Meta-analysis Of Observational Studies in Epidemiology (MOOSE) reporting proposals [45], and methods outlined in the Cochrane Handbook for Systematic Reviews of Interventions [46].

### Inclusion Criteria

#### Types of Studies

This review will include randomized controlled trials, cluster randomized controlled trials, and nonrandomized studies (NRS). NRS have been defined as quantitative studies estimating the effectiveness of an intervention (harm or benefit) that does not use randomization to allocate units to comparison groups [47]. We will include prospective cohort studies, case-control studies, controlled before-and-after studies, interrupted-time-series studies, and controlled trials with inappropriate randomization (quasiexperimental studies) [48-50]. We will consider publications in English, French, German, and Portuguese.

#### Types of Participants

This review will consider studies involving registered HCPs, including those with bachelor’s, master’s, or doctoral degrees in physiotherapy (PTs) and nursing (registered nurses [RNs], NPs) and who are delivering primary health care, including nursing and physiotherapy students. Physical therapists and PTs will be considered synonymous.

#### Types of Primary Health Care

We will include all types of primary health care settings such as private practices, community and health maintenance organization practices, community and private primary health care settings, hospital outpatient departments, practices in hospital settings, and hybrid primary health care practices including community and private practices, health maintenance organizations, and outpatient departments.

#### Types of Interventions

We will examine all types of educational interventions aimed at improving the EBP delivered by RNs, NPs, and PTs to adults living at home as part of active primary health care.

Based on the Cochrane Effective Practice and Organization of Care taxonomy of interventions [51], we will consider educational interventions targeting health care organizations and health care professionals (Textbox 1). We will exclude interventions targeting the regulatory, economic, or financial aspects of EBP.

**Types of educational interventions targeted at health care organizations and health care professionals.**

**Health care organizations**

- Ex-cathedra, interactive, online, or individual educational sessions on the steps and components of evidence-based practice (EBP) for registered nurses (RNs), nurse practitioners (NPs), and physiotherapists (PTs), such as reflexive practice, PICOT (population/patient problem; intervention; comparison; outcome; time)/PEO (population, patient, or problem; exposure; outcomes or themes) questions, critical appraisal of literature, and systematic reviews
- Organized journal clubs
- Systematic reviews organized within health care institutions

**Health care providers**

- Educational meetings aimed at RNs, NPs, and PTs alone or in collaboration with other health care professionals
- Distribution of educational materials (distribution of published or printed recommendations for clinical care, including clinical practice guidelines, audiovisual materials, and electronic publications)
- Web seminars and other individual-oriented educational activities, case studies, grand rounds, and mentoring
- Educational meetings (health care providers [HCPs] who have participated in conferences, lectures, workshops, or traineeships)
- Educational outreach visits (use of a trained person who has met with HCPs in their practice settings to give them information with the intent of changing their practice; information given may have included feedback on the HCP’s performance
- Patient-mediated interventions (new clinical information, not previously available, collected directly from patients and given to the HCP [eg, depression scores from an instrument])
- Educational games as an educational strategy to improve standards of care
- Interprofessional education meetings
- Audit and feedback (any summary of the clinical performance of health care over a specified period; it may also have included recommendations for clinical action; information may have been obtained from medical records, computerized databases, or the observation of patients)

#### Types of Outcome Measures

The review’s primary outcome measures will be increased or decreased beliefs, knowledge, implementation, and integration of EBP among RNs, NPs, and PTs active in primary health care settings (measured using methods [52,53] such as questionnaires, interviews, chart analysis, and self-reporting by RNs, NPs and PTs [53]), with a focus on dichotomous (yes/no), ordinal or continuous beliefs, and implementation or integration rates or scores.

The review’s secondary outcome measures will be the production of systematic reviews; numbers of journal clubs organized; numbers of grand rounds organized; development of EBP guidelines or practice guidelines for care or case management; and the implementation of EBP programs, mentor coaching, or tutorial programs.

#### Search Methods for the Identification of Studies

In collaboration with the medical librarians (MS and PM) and using predefined search terms, we will conduct a systematic literature search for published articles in the following electronic databases, from inception until October 31, 2020: Medline Ovid SP (from 1946), PubMed (NOT Medline[sb]; from 1996), Embase.com (from 1947), CINAHL Ebesco (from 1937), the Cochrane Central Register of Controlled Trials Wiley (from 1992), PsycINFO Ovid SP (from 1806), Web of Science Core collection (from 1900), PEDro (from 1999), the JBI Database of Systematic Reviews and Implementation Reports (from 1998), and the Trip Database (from 1997). We will also conduct a hand search of the bibliographies of all the relevant articles and a search for unpublished studies using Google Scholar, ProQuest Dissertations and Theses dissemination, Mednar, and WorldCat. The search will be completed by exploring the grey literature in OpenGrey and the Grey Literature Report from inception until October 31, 2020.

The search syntax of the included databases will serve as the basis for all search strategies, using descriptors (EMTREE and Medical Subject Headings [MeSH]) and text terms with Boolean operators “AND” and “OR.” The syntax consists of 4 search themes intersected by the Boolean terms “AND” and “OR.” The descriptor terms included in the health occupations of RNs, NPs, and the allied health occupations of PTs are described in Textbox 2, and descriptor terms and keywords included in the search strategy for educational interventions on EBP are described in Textbox 3.

**The 4 search themes in the search for evidence-based practice (EBP) for health occupations of registered nurses (RNs), nurse practitioners (NPs), and the allied health occupations of physiotherapists (PTs).**

**Terms for nurses (RNs and NPs) active in primary care**

- “Advanced Practice Nursing”
- “Nurse Practitioner”
- “Family Nurse Practitioner”
- “Community Health Nursing”
- “Home Health Nursing”
- “Parish Nursing”
- “Family Nursing”
- “Geriatric Nursing”
- “Hospice and Palliative Care Nursing”
- “Occupational Health Nursing”
- “Psychiatric Nursing”
- “Public Health Nursing”
- “Radiology and Imaging Nursing”
- “Rehabilitation Nursing”
- “Rural Nursing”
- “School Nursing”

**Terms related to evidence-based practice**

- “Evidence-based Healthcare”
- “Evidence-based Health Care”
- “Evidence-based Medicine”
- “Evidence-based Emergency Medicine”
- “Evidence-based Nursing”
- “Evidence-based Physical Therapy”
- “Evidence-based Physiotherapy”

**Terms for physiotherapy or physical therapy**

- “Physical Therapist”
- “Physiotherapists”
- “Evidence-based Physiotherapy“
- ”Evidence-based Physical Therapy”

**Terms related to evidence-based practice for physiotherapy or physical therapy**

- “Physical Therapy Specialty”
- “Physiotherapy Specialty”

**Descriptor terms and keywords included in the search strategy for educational interventions on EBP.**

**Education intervention–related descriptor terms**

- “Education, Nursing, Continuing”
- “Education, Nursing, Diploma Programmes”
- “Education, Nursing, Graduate”

**Education intervention–related keywords**

- “Mentoring”
- “Coaching”
- “Training Programme”
- “Workshops”

In addition to searching electronic databases, we will conduct a hand search of the bibliographies of all relevant articles and search for unpublished studies. We will consider publications in English, French, German, and Portuguese. Multimedia Appendix 1 presents the syntax used in all selected databases.

### Data Collection and Analysis

#### Study Selection

Two pairs of reviewers (HV and PM, RH and MS) will independently screen the titles and abstracts identified in searches in order to assess which studies meet the inclusion criteria. Disagreements will be resolved through discussion, or, if needed, a consensus will be reached after discussion with the co-authors (AGM and FP).

Two pairs of reviewers (HV and PM, RH and MS) will independently assess the full-text articles to ensure that they meet the inclusion criteria. Disagreements will be discussed and resolved with the co-authors (AGM and FP). A flowchart of the trial selection process has been drawn in accordance with the PRISMA-P statement [44] (Multimedia Appendix 2).

#### Data Extraction

Data extraction will be conducted independently by 2 pairs of authors (HV, RH, FP) using a specially designed, standardized data extraction form (Multimedia Appendix 3). Discrepancies will be resolved through discussion and consultation with the co-authors (FP, RH, FP).

The following information will be extracted from each included study: (1) study authors, year of publication, and country where the study was conducted; (2) study characteristics (including setting and design, duration of follow-up, and sample size); (3) participants’ characteristics (eg, profession, employment [% vs hours/week], employer, sex, age); (4) characteristics of interventions (eg, description and frequency of educational interventions, health care professionals involved); (5) characteristics of usual care group; and (6) types of outcome measures (Multimedia Appendix 3).

#### Assessment of the Risks of Bias in Included Studies

Two reviewers (HV and RH) will independently assess the risks of bias in all the randomized and nonrandomized studies of interventions (NRSIs) included. Disagreements will be resolved through discussion and consultation with the co-authors (HV, RH, FP).

We will use the validated Cochrane Risk of Bias Tool, version 2.0 [54], to assess the risk of bias in randomized trials and nonrandomized studies. This is based on 5 domains: (1) bias arising from the randomization process, (2) bias due to deviations from intended interventions, (3) bias due to missing outcome data, (4) bias in the measurement of the outcome, and (5) bias in the selection of the reported result. Each of these 5 domains will be rated as one of the following: (1) low risk of bias, (2) some concerns, or (3) high risk of bias. Declaring that a study has a particular level of risk of bias in any individual domain will mean that the study as a whole has a risk of bias.

We will use the validated Robins-I tool for assessing the risk of bias in NRSIs [55]. This tool covers 2 dimensions and 7 domains through which bias might be introduced into an NRSI: (1) pre-intervention and at intervention (bias due to confounding, bias in the selection of study participants, and bias in the classification of the intervention) and (2) post-intervention (bias due to deviations from intended interventions, bias due to missing data, bias in the measurement of outcomes, and bias in selection of the reported result) [55]. Any disagreements in quality assessments will be resolved through discussion.

### Statistical Analyses

Statistical analyses will be conducted following the recommendations of the Cochrane Handbook for Systematic Reviews of Interventions [46] and the PRISMA and MOOSE statements [56].

For dichotomous outcomes, average intervention effects will be calculated as relative risks with 95% CIs using a random-effects model [57]. For continuous data, a random-effects model will be used to calculate weighted mean differences with 95% CIs. If required, we will calculate standard deviations from the standard errors or 95% CIs presented in the articles. Heterogeneity will be quantified using the I^2^ and chi-squared tests. Funnel plots will be drawn, and Egger tests will be computed to explore the possibility of publication bias [58].

Reasons for heterogeneity in effect estimates will be sought in meta-analyses [59,60]. To explore the possible determinants of heterogeneity, we will conduct subgroup analyses according to selected study characteristics (eg, participants’ ages, country where the study was conducted, types of professions, types of interventions). Furthermore, sensitivity analyses will be conducted by (1) excluding relatively small studies (with fewer than 20 participants per randomization group) and (2) restricting the analyses to studies of good quality. Data will be analyzed using SPSS software (version 25.0) and Review Manager 5.3.

## Results

The search strategy retrieved a total of 18,299 references (16,795 from databases and 1504 from other sources), and after removing duplicates, we included 12,948 references (11,469 from databases and 1479 from other sources) that will be analyzed on the titles and abstracts by 2 independent researchers (Table 1). In the second phase, full-text papers will be retrieved from the references and analyzed based on the inclusion and exclusion criteria. Finally, all included full-text articles meeting the criteria will be analyzed and reported in a structured paper. The final results are expected in March 2021.

**Table 1:** Number of references retrieved with the search strategy.

Sources			Date of search		Number of references		
					Found in total		After removing duplicates
**Databases**							
	Medline OVID SP	October 31, 2020		3364		3356	
	Embase.com	October 31, 2020		4688		2718	
	PubMed	October 31, 2020		1749		1423	
	CINAHL EBSCO	October 31, 2020		3121		2120	
	PsycINFO OVID SP	October 31, 2020		1006		656	
	Cochrane Library Wiley	October 31, 2020		659		344	
	Web of Science – Core collection	October 31, 2020		2195		839	
	JBI OVID SP	October 31, 2020		13		13	
**Other Sources**							
	DART-Europe.eu	October 31, 2020		94		87	
	ProQuest Dissertations and Theses	October 31, 2020		377		359	
	SantéPsy	October 31, 2020		123		123	
	Lissa.fr	October 31, 2020		18		18	
	Opengrey.eu	October 31, 2020		93		93	
	PEDro.org	October 31, 2020		767		767	
	TRIP database.com	October 31, 2020		32		32	

## Discussion

Providing the best available, safe, high-quality health care is the gold standard objective in all health care settings. To the best of our knowledge, there exists no review of the effectiveness of educational interventions to increase the implementation of EBP among nurses and PTs working in primary health care. This systematic review research project will assess educational interventions aimed at both health care organizations and professional health care providers (RNs, NPs, and PTs). It will provide valuable information to HCPs, policymakers, and other stakeholders involved in primary health care.

## Appendix

Multimedia Appendix 1 Syntax of the systematic review.

Multimedia Appendix 2 PRISMA-P flowchart.

Multimedia Appendix 3 Data extraction sheet.

## Abbreviations

EBP: evidence-based practice
HCP: health care provider
MeSH: medical subject heading
NP: nurse practitioner
NRS: nonrandomized study
NRSI: nonrandomized studies of intervention
PICOT: population/patient problem; intervention; comparison; outcome; time
PEO: population, patient or problem; exposure; outcomes or themes
PRISMA-P: Preferred Reporting Items for Systematic Reviews and Meta-Analyses for Protocols
PT: physiotherapist
RN: registered nurse
