# Correction: Effectiveness of Educational Interventions to Increase Knowledge of Evidence-Based Practice Among Nurses and Physiotherapists in Primary Health Care: Protocol for a Systematic Review

**DOI:** 10.2196/27092

**Published:** 2021-01-15

**Authors:** Henk Verloo, Pauline Melly, Roger Hilfiker, Filipa Pereira

**Affiliations:** 1 Service of Old Age Psychiatry, University Hospital of Lausanne Sion Switzerland; 2 School of Health Sciences HES-SO Valais-Wallis Sion Switzerland; 3 Institute of Biomedical Sciences Abel Salazar, University of Porto Porto Portugal

In “Effectiveness of Educational Interventions to Increase Knowledge of Evidence-Based Practice Among Nurses and Physiotherapists in Primary Health Care: Protocol for a Systematic Review“ (J Med Internet Res 2020;9(11):e17621) four errors were noted.

Affiliation 2, applying to authors Henk Verloo, Pauline Melly, Roger Hilfiker, and Filipa Pereira was incorrectly listed as:

School of Health Sciences, Haute Ecole Spécialisé Suisse Occidentale Valais/Wallis, Sion, Switzerland

The correct affiliation for these authors is:

School of Health Sciences, HES-SO Valais-Wallis, Sion, Switzerland

In the originally published manuscript, degree information for author Pauline Melly was listed as "MAS", and degrees for authors Roger Hilfiker and Filipa Pereira were listed as "DPhil". As these degrees have not yet been completed, all have been removed from the corrected manuscript. For author Filipa Pereira, the degree "MSc" has also been added to the corrected manuscript.

The correction will appear in the online version of the paper on the JMIR Publications website on January 15, 2021, together with the publication of this correction notice. Because this was made after submission to PubMed, PubMed Central, and other full-text repositories, the corrected article has also been resubmitted to those repositories.

