# Supplementary material for: Effectiveness of Educational Interventions to Increase Knowledge of Evidence-Based Practice Among Nurses and Physiotherapists in Primary Health Care: Protocol for a Systematic Review
Source: JMIR Res Protoc. 2020 Nov 2;9(11):e17621. doi: 10.2196/17621 (PMC7669447; doi:10.2196/17621)
Supplement: Multimedia Appendix 1 [file resprot_v9i11e17621_app1.docx]

**Recherche systématique de littérature**

Effectiveness of educational interventions to increase the implementation of evidence-based practice in daily practice among nurses and physiotherapists in primary healthcare: a protocol for a systematic review and meta-analysis

**Recherche effectuée par** :

Pauline MELLY, School of Health Sciences, HES-SO Valais-Wallis, 5, Chemin de l’Agasse, CH-1950 Sion, Switzerland

**Recherche révisée par** :

Joëlle ROSSELET AMOUSSOU, Psychiatry library, Education and Research Department, Lausanne University Hospital and University of Lausanne, Site de Cery, 1008 Prilly, Lausanne, Switzerland.

**Date** : 11.octobre 2019

**A l’attention de** :

Henk VERLOO, School of Health Sciences, HES-SO Valais-Wallis, 5, Chemin de l’Agasse, CH-1950 Sion, Switzerland

# Question de recherche

Effectiveness of educational interventions to increase the implementation of evidence-based practice in daily practice among nurses and physiotherapists in primary healthcare: a protocol for a systematic review and meta-analysis

**PROSPERO REGISTRATION NUMBER**: CRD42017077309

# Recherche de revues systématiques

## Epistomonikos

| **Stratégie** | (nurs* OR psych*) AND "primary care" AND "evidence based" AND (education* OR training) |
| --- | --- |
| **Commentaires** | Filtre Publication type : Systematic review |
| **Réf. trouvée.** | 44 références trouvées le 17 mai 2019 |

# Sources de données exploitées

**Bases de données principales**

Embase.com

Medline Ovid SP

Pubmed

CINAHL EBSCO

PsycINFO Ovid SP

Web of Science – Core collection

Cochrane library Wiley

JBI OVID SP

**Thèses**

DART-Europe.eu

ProQuest Dissertations and Theses

**Littérature grise**

Opengrey.eu

**Autres bases de données**

TRIP database.com

SantéPsy

Lissa.fr

PEDro.org

# Vocabulaire

| **Concepts choisis** | Nurse / physiotherapist | Primary care | EBP |
| --- | --- | --- | --- |
| **Mots libres**  Syntaxe Embase | nurs* OR physiotherap* OR ((physical OR physio) NEAR/3 (therapy OR therapist* OR therapies OR treatment*)) | (outpatient* OR homecare OR ((famil* OR general) NEAR/3 (practice* OR medicine)) OR ((primary OR communit* OR ambulator* OR home) NEAR/3 (care OR healthcare OR practice* OR "health care")) | EBP OR EBM OR EBN OR (evidence NEAR/3 (adopt* OR application* OR apply* OR implement* OR practice* OR uptake OR utilis* OR utiliz*)) OR "evidence based" OR evidence-informed |
| **Emtree** | 'nurse'/exp OR 'nursing student'/exp OR 'nursing'/exp OR 'physiotherapist'/exp OR 'physiotherapy'/de OR ‘home physiotherapy’/de OR 'physical therapy student'/exp | 'primary health care'/exp OR 'general practice'/exp OR 'ambulatory care'/exp OR 'outpatient department'/exp OR 'community care'/exp OR 'home care'/exp OR 'family medicine'/exp | ‘evidence based practice’/de OR ‘evidence based medicine’/de OR ‘evidence based nursing’/de |
| **MeSH** | "Nurses"[Mesh] OR "Students, Nursing"[Mesh] OR "Physical Therapy Specialty"[Mesh] OR "Physical Therapists"[Mesh] OR "Physical Therapy Modalities"[Mesh] | "Primary Health Care"[Mesh] OR "General Practice"[Mesh] OR "Ambulatory Care"[Mesh] OR "Community Health Nursing"[Mesh] OR "Home Care Services"[Mesh] | "Evidence-Based Practice"[Mesh] |
| **CINAHL** | MH "Nurses+" OR MH "Students, Nursing+" OR MH "Nursing Care+" OR MH "Physical Therapy+" OR MH "Students, Physical Therapy" OR MH "Physical Therapists" OR MH "Physical Therapy Practice" | MH "Primary Health Care" OR MH "Family Practice" OR MH "Ambulatory Care" OR MH "Ambulatory Care Nursing" OR MH "Ambulatory Care Facilities+" OR MH "Outpatient Service" OR MH "Community Health Nursing+" OR MH "Home Health Care+" | MH "Professional Practice, Evidence-Based+" |
| **PsycInfo** | exp Nurses/ OR Students, Nursing/ OR exp Nursing/ OR exp physical therapists/ OR physical therapy/ OR Physical Treatment Methods/ | primary health care/ OR exp outpatient treatment/ OR home care/ OR family medicine/ | evidence based practice/ |

## Commentaires sur les concepts

### Educational intervention

Il a été décidé de ne pas conserver le 4^e^ concept d'intervention éducationnelle afin d'avoir des articles qui parlent de l'implémentation de l'EBP en général, qui peut se traduire par des interventions, des outils, des canaux d'information.

# Stratégies de recherche pour les bases de données

## Bases de données principales

### Embase.com

| **Stratégie** | ('nurse'/exp OR 'nursing student'/exp OR 'nursing'/exp OR 'physiotherapist'/exp OR 'physiotherapy'/de OR 'home physiotherapy'/de OR 'physical therapy student'/exp OR (nurs* OR physiotherap* OR ((physical OR physio) NEAR/3 (therapy OR therapist* OR therapies OR treatment*))):ab,ti,kw) **AND** ('primary health care'/exp OR 'general practice'/exp OR 'ambulatory care'/exp OR 'family medicine'/exp OR 'outpatient department'/exp OR 'community care'/exp OR 'home care'/exp OR (outpatient* OR homecare OR ((famil* OR general) NEAR/3 (practice* OR medicine)) OR ((primary OR communit* OR ambulator* OR home) NEAR/3 (care OR healthcare OR practice* OR "health care"))):ab,ti,kw) **AND** ('evidence based practice'/de OR 'evidence based medicine'/de OR 'evidence based nursing'/de OR (EBP OR EBM OR EBN OR (evidence NEAR/3 (adopt* OR application* OR apply* OR implement* OR practice* OR uptake OR utilis* OR utiliz*)) OR "evidence based" OR evidence-informed):ab,ti,kw) |
| --- | --- |
| **Commentaires** |  |
| **Réf. trouvée.** | 4688 références trouvées le 09.10.2019 |

### Medline Ovid SP

| **Stratégie** | (exp Nurses/ OR Students, Nursing/ OR exp Nursing/ OR Physical Therapists/ OR Physical Therapy Specialty/ OR Physical Therapy Modalities/ OR (nurs* OR physiotherap* OR ((physical OR physio) ADJ3 (therapy OR therapist* OR therapies OR treatment*))).ab,ti,kf.) **AND** (exp Primary Health Care/ OR exp General Practice/ OR exp Ambulatory Care/ OR exp Community Health Nursing/ OR exp Home Care Services/ OR (outpatient* OR homecare OR ((famil* OR general) ADJ3 (practice* OR medicine)) OR ((primary OR communit* OR ambulator* OR home) ADJ3 (care OR healthcare OR practice* OR "health care"))).ab,ti,kf.) **AND** (evidence-based practice/ OR exp evidence-based medicine/ OR evidence-based nursing/ OR (EBP OR EBM OR EBN OR (evidence ADJ3 (adopt* OR application* OR apply* OR implement* OR practice* OR uptake OR utilis* OR utiliz*)) OR "evidence based" OR evidence-informed).ab,ti,kf.) |
| --- | --- |
| **Commentaires** | Ovid MEDLINE(R) and Epub Ahead of Print, In-Process & Other Non-Indexed Citations and Daily 1946 to October 08, 2019 |
| **Réf. trouvée.** | 3364 références trouvées le 09.10.2019 |

### Pubmed (NOT Medline[sb])

| **Stratégie** | (nurs*[tiab] OR physiotherap*[tiab] OR ((physical[tiab] OR physio[tiab]) AND (therapy[tiab] OR therapist*[tiab] OR therapies[tiab] OR treatment*[tiab]))) **AND** (((famil*[tiab] OR general[tiab]) AND (practice*[tiab] OR medicine[tiab])) OR ((primary[tiab] OR communit*[tiab] OR ambulator*[tiab] OR home[tiab]) AND (care[tiab] OR healthcare[tiab] OR practice*[tiab] OR "health care"[tiab])) OR outpatient*[tiab] OR homecare[tiab]) **AND** (EBP[tiab] OR EBM[tiab] OR EBN[tiab] OR (evidence[tiab] AND (adopt*[tiab] OR application*[tiab] OR apply*[tiab] OR implement*[tiab] OR practice*[tiab] OR uptake[tiab] OR utilis*[tiab] OR utiliz*[tiab])) OR "evidence based"[tiab] OR evidence-informed[tiab]) **NOT** medline[sb] |
| --- | --- |
| **Commentaires** | Limitation aux recherches non indexées pour Medline : **NOT** medline[sb] |
| **Réf. trouvée.** | 1749 références trouvées le 09.10.2019 |

### CINAHL EBSCO

| **Stratégie** | (MH "Nurses+" OR MH "Students, Nursing+" OR MH "Nursing Care+" OR MH "Physical Therapy+" OR MH "Students, Physical Therapy" OR MH "Physical Therapists" OR MH "Physical Therapy Practice" OR TI (nurs* OR physiotherap* OR ((physical OR physio) W4 (therapy OR therapist* OR therapies OR treatment*))) OR AB (nurs* OR physiotherap* OR ((physical OR physio) W4 (therapy OR therapist* OR therapies OR treatment*)))) **AND** (MH "Primary Health Care" OR MH "Family Practice" OR MH "Ambulatory Care" OR MH "Ambulatory Care Nursing" OR MH "Ambulatory Care Facilities+" OR MH "Outpatient Service" OR MH "Community Health Nursing+" OR MH "Home Health Care+" OR TI (outpatient* OR homecare OR ((famil* OR general) W4 (practice* OR medicine)) OR ((primary OR communit* OR ambulator* OR home) W4 (care OR healthcare OR practice* OR "health care"))) OR AB (outpatient* OR homecare OR ((famil* OR general) W4 (practice* OR medicine)) OR ((primary OR communit* OR ambulator* OR home) W4 (care OR healthcare OR practice* OR "health care")))) **AND** (MH "Professional Practice, Evidence-Based+" OR TI (EBP OR EBM OR EBN OR (evidence W4 (adopt* OR application* OR apply* OR implement* OR practice* OR uptake OR utilis* OR utiliz*)) OR "evidence based" OR evidence-informed) OR AB (EBP OR EBM OR EBN OR (evidence W4 (adopt* OR application* OR apply* OR implement* OR practice* OR uptake OR utilis* OR utiliz*)) OR "evidence based" OR evidence-informed)) |
| --- | --- |
| **Commentaires** |  |
| **Réf. trouvée.** | 3121 références trouvées le 09.10.2019 |

### PsychINFO Ovid SP

| **Stratégie** | (exp Nursing/ OR exp Nurses/ OR exp Nursing Students/ OR physical therapists/ OR physical therapy/ OR exp physical treatment methods/ OR (nurs* OR physiotherap* OR ((physical OR physio) ADJ3 (therapy OR therapist* OR therapies OR treatment*))).ab,ti.) **AND** (primary health care/ OR exp outpatient treatment/ OR home care/ OR family medicine/ OR (outpatient* OR homecare OR ((famil* OR general) ADJ3 (practice* OR medicine)) OR ((primary OR communit* OR ambulator* OR home) ADJ3 (care OR healthcare OR practice* OR "health care"))).ab,ti.) **AND** (exp evidence-based practice/ OR exp best practices/ OR (EBP OR EBM OR EBN OR (evidence ADJ3 (adopt* OR application* OR apply* OR implement* OR practice* OR uptake OR utilis* OR utiliz*)) OR "evidence based" OR evidence-informed).ab,ti.) |
| --- | --- |
| **Commentaires** | PsycINFO 1806 to September Week 5 2019 |
| **Réf. trouvée.** | 1006 références trouvées le 09.10.2019 |

### Web of Science -Core collection

| **Stratégie** | **TS=**(nurs* OR physiotherap* OR (("physical" OR "physio") NEAR/4 ("therapy" OR therapist* OR "therapies" OR treatment*))) **AND** **TS=**(outpatient* OR homecare OR ((famil* OR general) NEAR/4 (practice* OR medicine)) OR (("primary" OR communit* OR ambulator* OR "home") NEAR/4 ("care" OR "healthcare" OR practice* OR "health care"))) **AND** **TS=**("EBP" OR "EBM" OR "EBN" OR (evidence NEAR/4 (adopt* OR application* OR apply* OR implement* OR practice* OR "uptake" OR utilis* OR utiliz*)) OR "evidence based" OR "evidence-informed") |
| --- | --- |
| **Commentaires** |  |
| **Réf. trouvée.** | 2196 références trouvées le 09.10.2019 |

### Cochrane library Wiley

| **Stratégie** | (nurs* OR physiotherap* OR ((physical OR physio) NEAR/3 (therapy OR therapist* OR therapies OR treatment*)))**:ab,ti,kw AND** (outpatient* OR homecare OR ((famil* OR general) NEAR/3 (practice* OR medicine)) OR ((primary OR communit* OR ambulator* OR home) NEAR/3 (care OR healthcare OR practice* OR "health care")))**:ab,ti,kw** **AND** (EBP OR EBM OR EBN OR (evidence NEAR/3 (adopt* OR application* OR apply* OR implement* OR practice* OR "uptake" OR utilis* OR utiliz*)) OR "evidence based" OR evidence-informed)**:ab,ti,kw** |
| --- | --- |
| **Commentaires** | **(22 reviews, 637 trials, 1 éditorial)** |
| **Réf. trouvée.** | 660 références trouvées le 09.10.2019 |

### JBI OVID SP

| **Stratégie** | (nurs* OR physiotherap* OR ((physical OR physio) ADJ4 (therapy OR therapist* OR therapies OR treatment*)))**.ti,hw,tx. AND** (outpatient* OR homecare OR ((famil* OR general) ADJ4 (practice* OR medicine)) OR ((primary OR communit* OR ambulator* OR home) ADJ4 (care OR healthcare OR practice* OR "health care")))**.ti,hw,tx. AND** (EBP OR EBM OR EBN OR (evidence ADJ4 (adopt* OR application* OR apply* OR implement* OR practice* OR uptake OR utilis* OR utiliz*)) OR "evidence based" OR evidence-informed)**.ti.** |
| --- | --- |
| **Commentaires** |  |
| **Réf. trouvée.** | 13 références trouvées le 09.10.2019 |

## Thèses

DART-Europe.eu

| **Stratégie** | (nurs* OR physiotherap* OR "physical therapy" OR "physical therapies" OR "physio therapy" OR "physio therapies") **AND** (outpatient* OR "family practice" OR "family medicine" OR "general practice" OR "general practices" OR "Primary care" OR "primary healthcare" OR "primary health care" OR "community care" OR "community health care" OR "home care" OR homecare OR "home health care" OR "ambulatory care") **AND** (EBP OR EBM OR EBN OR evidence) |
| --- | --- |
| **Commentaires** |  |
| **Réf. trouvée.** | 94 références trouvées le 09.10.2019 |

### ProQuest Dissertations and Theses

| **Stratégie** | **AB,TI**(nurs* OR physiotherap* OR "physical therapy" OR "physical therapies" OR "physio therapy" OR "physio therapies") AND **AB,TI**(outpatient* OR "family practice" OR "family medicine" OR "general practice" OR "general practices" OR "Primary care" OR "primary healthcare" OR "primary health care" OR "community care" OR "community health care" OR "home care" OR homecare OR "home health care" OR "ambulatory care") AND **AB,TI**(EBP OR EBM OR EBN OR "evidence based" OR "evidence-informed" OR (evidence AND (adopt* OR application* OR apply* OR implement* OR practice* OR uptake OR utilis* OR utiliz*))) |
| --- | --- |
| **Commentaires** | **Filtre : thèses de doctorat** |
| **Réf. trouvée.** | 377 références trouvées le 09.10.2019 |

## Littérature grise

### Opengrey.eu

| **Stratégie** | (nurs* OR physiotherap* OR ((physical OR physio) AND (therapy OR therapist* OR therapies OR treatment*))) AND ((famil* AND (practice* OR medicine)) OR ((primary OR communit* OR ambulator* OR home) AND (care OR healthcare OR practice* OR "health care"))) AND (EBP OR EBM OR EBN OR (evidence AND (adopt* OR application* OR apply* OR implement* OR practice* OR uptake OR utilis* OR utiliz*)) OR evidence based OR evidence-informed) |
| --- | --- |
| **Commentaires** |  |
| **Réf. trouvée.** | 93 références trouvées le 09.10.2019 – **pas de Endnote** |

## Autres bases de données

### TRIP database

| **Stratégie** | **title:(**(nurs* OR physiotherap* OR "physical therapy" OR "physical therapies" OR "physio therapy" OR "physio therapies") **AND** (outpatient* OR "family practice" OR "family medicine" OR "general practice" OR "general practices" OR "Primary care" OR "primary healthcare" OR "primary health care" OR "community care" OR "community health care" OR "home care" OR homecare OR "home health care" OR "ambulatory care") **AND** ((nurs* OR physiotherap* OR ((physical OR physio) AND (therapy OR therapist* OR therapies OR treatment*))) AND ((famil* AND (practice* OR medicine)) OR ((primary OR communit* OR ambulator* OR home) AND (care OR healthcare OR practice* OR "health care"))) **AND** (EBP OR EBM OR EBN OR (evidence AND (adopt* OR application* OR apply* OR implement* OR practice* OR uptake OR utilis* OR utiliz*)) OR evidence based OR evidence-informed)**)** |
| --- | --- |
| **Commentaires** |  |
| **Réf. trouvée.** | 32 références trouvées le 09.10.2019 – **pas de Endnote** |

### Santé Psy

| **Stratégie** | Recherche "evidence based" (122 références)  Recherche "médecine factuelle" (99 références) |
| --- | --- |
| **Commentaires** |  |
| **Réf. trouvée.** | 123 références trouvées le 09.10.2019 |

### Lissa

| **Stratégie** | ((soins infirmiers.mc OU infirmières et infirmiers.mc OU kinésithérapeutes.mc OU kinésithérapie (spécialité).mc OU infirmier*.tl OU physio*.tl) ET (médecine générale.mc OU soins de santé primaires.mc OU soins ambulatoires.mc OU patients en consultation externe.mc OU services de santé communautaires.mc OU services de soins à domicile.mc OU médecine de famille.mc OU premier recours.tl OU première ligne de soins.tl OU ambulatoire.tl OU médecine général*.tl OU proximité.tl OU médecine famil*.tl) ET (soins infirmiers factuels.mc OU médecine factuelle.mc OU pratique factuelle.mc OU médecine fondée sur les faits.mc OU soins infirmiers factuels.tl OU médecine factuelle.tl OU pratique factuelle.tl OU médecine fondée sur les faits.tl OU données probantes.tl OU EBM.tl OU EBP.tl)) |
| --- | --- |
| **Commentaires** |  |
| **Réf. trouvée.** | 18 références trouvées le 09.10.2019 |

### PEDro.org

| **Stratégie** | "primary care" |
| --- | --- |
| **Commentaires** | Filtre : clinical trial |
| **Réf. trouvée.** | 767 références trouvées le 18.10.2019 – **pas de Endnote** |

# Résultats

| **Base de données** | **Date de la recherche** | **Nombre de références…** | |
| --- | --- | --- | --- |
|  |  | …trouvées… | …et après dédoublonnage |
| Medline OVID SP | 09.10.2019 | 3364 | 3356 |
| Embase.com | 09.10.2019 | 4688 | 2718 |
| PubMed | 09.10.2019 | 1749 | 1423 |
| CINAHL EBSCO | 09.10.2019 | 3121 | 2120 |
| PsycINFO OVID SP | 09.10.2019 | 1006 | 656 |
| Cochrane Library Wiley | 09.10.2019 | 659 | 344 |
| Web of Science – Core collection | 09.10.2019 | 2195 | 839 |
| JBI OVID SP | 09.10.2019 | 13 | 13 |
| Total |  | 16795 | 11469 |

| **Autres ressources** | **Date de la recherche** | **Nombre de références…** | |
| --- | --- | --- | --- |
|  |  | …trouvées… | …et après dédoublonnage |
| DART-Europe.eu | 09.10.2019 | 94 | 87 |
| ProQuest Dissertations and Theses | 09.10.2019 | 377 | 359 |
| SantéPsy | 09.10.2019 | 123 | 123 |
| Lissa.fr | 09.10.2019 | 18 | 18 |
| Total |  | 612 | 587 |

**Ressources non intégrées dans Endnote**

| **Autres ressources** | **Date de la recherche** | **Nombre de références non dédoublonnées** |
| --- | --- | --- |
| Opengrey.eu | 09.10.2019 | 93 |
| PEDro.org | 18.10.2019 | 767 |
| TRIP database.com | 09.10.2019 | 32 |
| Total |  | 892 |
