# Supplementary material for: Effectiveness of Educational Interventions to Increase Knowledge of Evidence-Based Practice Among Nurses and Physiotherapists in Primary Health Care: Protocol for a Systematic Review
Source: JMIR Res Protoc. 2020 Nov 2;9(11):e17621. doi: 10.2196/17621 (PMC7669447; doi:10.2196/17621)
Supplement: Multimedia Appendix 2 [file resprot_v9i11e17621_app2.docx]

Additional records identified through other sources
(n = 1’504)

Records identified through database searching
(n = 16’795)

Records after duplicates removed
(n = 12’948)

Records excluded
(n = )

Records screened
(n = )

Full-text articles assessed for eligibility
(n = )

Full-text articles excluded, with reasons
(n = )

Studies included in qualitative synthesis
(n = )

Studies included in quantitative synthesis (meta-analysis)
(n = )
